# Luminescence and photocatalytic degradation of indigo carmine in the presence of Sm^3+^doped ZnS nanoparticles

**DOI:** 10.1038/s41598-023-49912-6

**Published:** 2023-12-17

**Authors:** Lal Lianmawii, N. Mohondas Singh

**Affiliations:** Department of Chemistry, Mizoram University, Tanhril, Aizawl, Mizoram 796004 India

**Keywords:** Chemistry, Materials science

## Abstract

Industrial wastewater discharge is well acknowledged to constitute a significant environmental and public health risk. In addition, synthetic dyes used in the textile sector are major culprits in water pollution. The amount of water polluted by these dyes is simply staggering. We urgently address this issue to protect our planet and health. The degradation of indigo carmine dye in the presence of Sm^3+^-doped ZnS nanoparticles is reported in this study and characterized by XRD, FTIR, SEM, EDX, TEM, BET, PL, UV, etc. The particle size calculated from the Scherrer equation was 3–12 nm. When excited at 395 nm, Sm^3+^ undergoes f–f transitions, which are visible as prominent peaks in the photoluminescence spectrum at 559, 595, and 642 nm wavelengths. The catalyst showed vigorous catalytic activity for dye degradation, with a 93% degradation rate when used at 15 mg/L catalyst within 210 min. The reaction was found to have pseudo-first-order kinetics. After applying the Freundlich and Langmuir data, the Langmuir isotherm offered the best fit. The findings indicate that the Sm^3+^-doped ZnS catalyst might be successfully used in the degradation of dyes present in the environment. Doping with Sm^3+^ ions can significantly change the photocatalytic breakdown of indigo carmine and the luminescence characteristics of ZnS.

## Introduction

Dyes and dye-related intermediates, surfactants, and minor metal residues are the chemical pollutants concerned with environmental preservation^1–3^. 10–15% of all dyes are lost to wastewater during dyeing. Therefore, the effluent must be treated before being released into the environment^4,5^. Dyes may remain in the water for an extended period, lowering the quality of the aquatic ecosystem^6^. Dyes have the potential to pollute the environment due to their chemical makeup, persistence, and negative environmental impacts. These pigments frequently resist both biological and physical processing methods. Industries, including textile, printing, and papermaking, frequently employ dyes. These companies pollute the water by discharging wastewater that contains dyes. Fish, algae, and other aquatic life may become poisoned by certain dyes and byproducts of their decomposition. Persistent and poisonous dyes may build up in the food chain. Animals that consume tainted food or water may collect toxic colour chemicals from humans and other higher-level predators^7,8^. Some colours may contain toxic heavy metals like chromium, lead, cadmium, or mercury that can bioaccumulate in the environment. Light penetration and photosynthetic activity decreased as a result of the dyes' severe toxicity and mutagenicity, which caused an oxygen deficit and restricted downstream beneficial applications, including recreation, drinking water, and irrigation. The textile industry uses toxic substances that harm the environment and people's health^9,10^. Advanced oxidation techniques (AOPs) have shown potential for removing dyes from wastewater^11^. Complete organic matter mineralization is achievable in the advanced oxidation process (AOPs)^12^. Fast reaction times and nonselective oxidation are AOP advantages that allow for treating several pollutants simultaneously^13^. These pollutants undergo a single breakdown by the OH radical before forming intermediates. As a result of their interaction with the oxidants, those intermediates transform into stable molecules. The main benefits of photocatalytic degradation include its low operating costs, high rate of target pollutant mineralization, and lack of need for high pressure and temperature during treatment. Therefore, the removal and degradation of dyes is a growing and important topic of research, which may encourage research efforts to reduce the environmental issues with textile dye effluents and safeguard the environment^14^. Additionally, doping semiconductor photocatalysts with a 4f. electron configuration in lanthanide ions might significantly increase the photocatalytic activity and raise the separation rate of photo-induced charge carriers^15,16^. Rare earth metals with vacant 4f. and 5d orbitals are widely used to facilitate catalysis or act as catalysts. Zinc sulfide is a substantial (II-VI) semiconductor with a significant direct band gap of 3.68 eV (bulk)^17^. Due to its numerous uses as phosphors and catalysts, ZnS has been researched. Due to its extreme thermal shock tolerance, low bulk losses, and stability in nearly all situations, ZnS is a potential material for solar cells, electroluminescent devices, and numerous other optoelectronic devices. Due to their potential to increase photocatalytic effectiveness, heterostructures containing zinc sulfide (ZnS) nanoparticles are a hot topic in photocatalysis. ZnS is a wide-bandgap semiconductor that makes it ideal for photocatalytic applications, including the development of hydrogen and the degradation of contaminants. A heterostructure's various parts can display various catalytic activities. For instance, ZnS can easily produce electron–hole pairs, whereas another substance can offer active sites for particular catalytic reactions. An increase in overall photocatalytic activity may result from this synergy. This raises the chances of the charges taking part in photocatalytic reactions and extends the lifetime of the charges^18,19^. Because the 3p orbital of the S atom makes up the valence band of metal sulfides, compared to metal oxides, they feature a complete valence band and a smaller band gap^20,21^. As a result, metal sulfides, which are visible light-responsive photocatalysts, particularly ZnS and CdS, have attracted much attention in photocatalysis research. TiO_2_ also functions as a semiconductor with a significant bandgap (3.0–3.7 eV) when exposed to light with 380 nm wavelengths, which leads to the formation of electron–hole pairs in redox reactions. When TiO_2_ is ignited, reactive oxygen species (ROS) are produced, and ROS have the power to oxidize a wide range of organic compounds in aqueous solutions. When TiO_2_ and near-UV light are present, these oxidation reactions may convert organic pollutants to carbon dioxide^22^. It is a fascinating option for photocatalytic applications due to its high surface-area-to-volume ratio. The increased surface area may address some of the drawbacks of ZnO and TiO_2_, which can improve photon absorption and enable more effective separation of photogenerated electron–hole pairs^23^.

In this study, ZnS activity, stability, or selectivity for dye degradation may all be enhanced by adding samarium (Sm^3+^). Analysis was also conducted on the effects of operating parameters on the degradation rate, such as pH, catalyst loading, and initial dye concentration. Zinc sulfide (ZnS) is a semiconductor substance that works well as a photocatalyst. ZnS has a large surface area, which makes more photon absorption sites possible. Light energy can be captured and used for photocatalytic reactions more effectively. The separation of photogenerated electron–hole pairs dramatically influences the efficiency of photocatalysts. By encouraging the separation of these charge carriers and lowering the likelihood of recombination, the increased surface area of ZnS can boost the total photocatalytic efficiency^24^. ZnS is a promising choice for a variety of photocatalytic applications, including environmental cleanup and solar energy conversion, due to its unique characteristics and high surface area, which may be able to address some of these restrictions. According to specific research, ZnS is an effective and prospective catalyst for the photocatalytic elimination of organic pollutants^16,25,26^. In order to eliminate toxic dyes that are present in the environment, this study will offer new strategies for researchers. As far as we know, research has yet to be published on the photocatalytic degradation of indigo carmine by Sm^3+^-doped ZnS nanoparticles. This paper introduces a straightforward reflux approach for synthesising Sm^3+^-doped ZnS nanoparticles.

## Results

**Figure 1:**
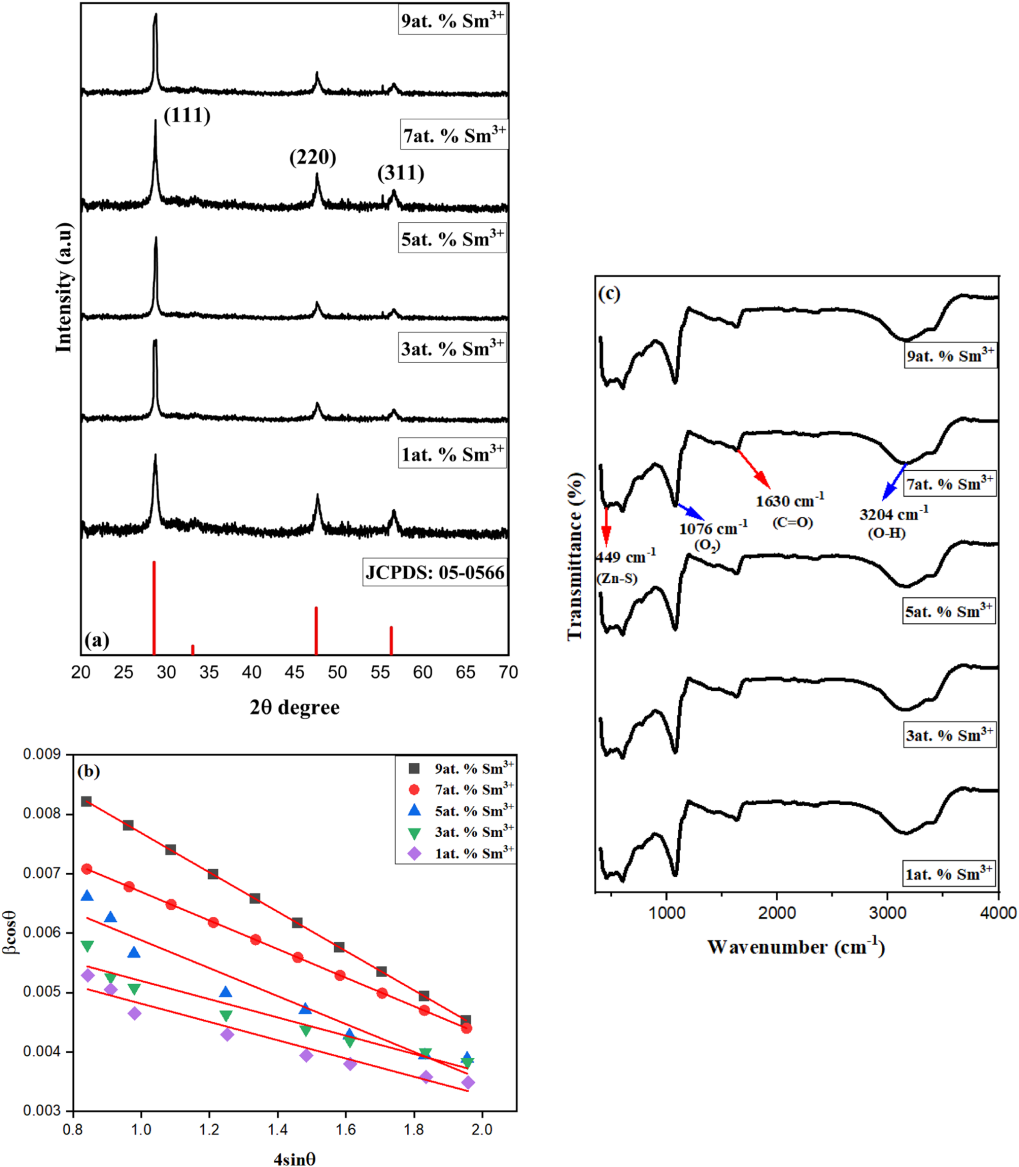
(**a**) XRD pattern of Sm^3+^-doped ZnS with different concentrations (1, 3, 5, 7, 9at. %). (**b**) Plot of 4 sinθ vs β_hkl_ cosθ of Sm^3+^-doped ZnS with different concentrations (1, 3, 5, 7, 9 at. %). (**c**) FTIR spectra of Sm^3+^-doped ZnS 1, 3, 5, 7, 9at. %.

Figure 1a confirms the XRD cubic zinc ZnS structure (JCPDS No. 05-0566) with the lattice parameters and volume given in Table 1. When the amounts of Sm^3+^ doping increased, the nanoparticle crystallinity also increased. The diffraction peaks of Sm^3+^-doped ZnS exhibit a slight shift towards higher 2θ values. The synthesized materials exhibited distinct diffraction peaks, demonstrating the sample crystallinity. Using Scherrer’s formula, the average particle size was calculated using the formula D = (Kλ)/β cosθ, where ‘K’ is constant (0.9) and the wavelength of X-rays employed ‘λ’ (1.5406 Å)^27^. According to the Scherrer equation, Sm^3+^-doped ZnS nanocrystals have a 3–12 nm particle size. Furthermore, the experimental results show that the broadening for small crystallites is distinct from the strain-induced broadening. Therefore, the full-width half maximum (FWHM) of the XRD pattern provided in the equation may be calculated using the linear sum of the FWHMs of size, strain, and instrument^28^:

1 $$\upbeta _{{{\text{tot}}}} = {\upbeta }_{{{\text{size}}}} +\upbeta _{{{\text{strain}}}} +\upbeta _{{{\text{instrument}}}}$$

**Table 1:** Comparison of standard values for Sm^3+^-doped ZnS their lattice parameters and volume.

Standard parameters	Standard values	1at. % Sm^3+^	3at. % Sm^3+^	5at. % Sm^3+^	7at. % Sm^3+^	9at. % Sm^3+^
Lattice parameter (a) Å	5.406	5.322	5.442	5.301	5.228	5.202
Lattice volume (Å^3^)	157.99	157.78	157.65	157.92	157.45	157.40

Here, the instrument broadening β_hkl_ was adjusted by each diffraction peak, and a Lorentzian profile was fitted. Strain broadening is the term used to describe the contribution of lattice strain to peak broadening, which is often caused by local deformation of the lattice. If the strain is taken to be constant throughout all crystallographic orientations and the crystal is isotropic, the strain broadening can be depicted as follows^29^:

2 $$\upvarepsilon =\upbeta _{{{\text{hkl}}}} /4\tan\uptheta .$$

Substituting values of β_size_ and β_strain_ in the above equation:

3 $$\upbeta _{{{\text{hkl}}}} = {\text{K}}\uplambda /{\text{D}}\cos\uptheta + 4\upvarepsilon .$$

The above Eq. (3) can be rearranged by:

4 $$\upbeta _{{{\text{hkl}}}} \cos\uptheta = {\text{K}}\uplambda /{\text{D}}\cos\uptheta + 4\upvarepsilon \sin\uptheta .$$

The parameter of the micro-strain is

5 $$\upvarepsilon = \Delta {\text{D}}_{{{\text{hkl}}}} /{\text{D}}_{{{\text{hkl}}}} .$$

A graph is drawn between 4 sinθ and β_hkl_ cosθ, as shown in Fig. 1b For the samples corresponding to 1, 3, 5, 7, and 9 at. % the micro-strain calculated from the graph is -0.0331, -0.0241, -0.0235, -0.0154, and -0.0164, respectively. The R^2^ values for different concentrations were also found to be 0.982, 0.973, 0.955, 0.942, and 0.933. Williamson-Hall plots of samples show a negative slope, which denotes the particle’s compressive strain.

Figure 1c displays the synthesized Sm^3+^-doped ZnS FTIR spectra at 1, 3, 5, 7, and 9at.%. This demonstrates the capability of infrared spectroscopy to recognize the sample's functional groups. IR spectroscopies, which depend on the absorption of infrared light, are used to determine the vibrational energy of molecules^30^. A massive peak of 3204 cm^−1^, which was most likely caused by the adsorptive moisture on the surfaces of the materials, was attributed to the stretching of the –OH. This peak may indicate adsorptive moisture on the material's surface since it indicates the existence of hydroxyl (alcohol) functional groups in the substance. The significant peaks detected around 1630 cm^−1^ can also be attributed to the carboxyl (C=O) stretching vibration because the functional group is present in the adsorbed residues^31^. This suggests that the adsorbed residues contain carboxylic acid or ester groups. The peak seen at 1076 cm^−1^ is caused by oxygen (O_2_). It can be a reference peak or show that the substance contains oxygen-containing functional groups. The usual ZnS primary bonding peak (Zn–S stretching or lattice vibrations) is seen at 449 cm^−1^. The primary bonding in ZnS is linked to the peak at this wavenumber. It is frequently related to lattice vibrations or Zn–S stretching and can be used to identify whether ZnS is present in the material.

**Figure 2:**
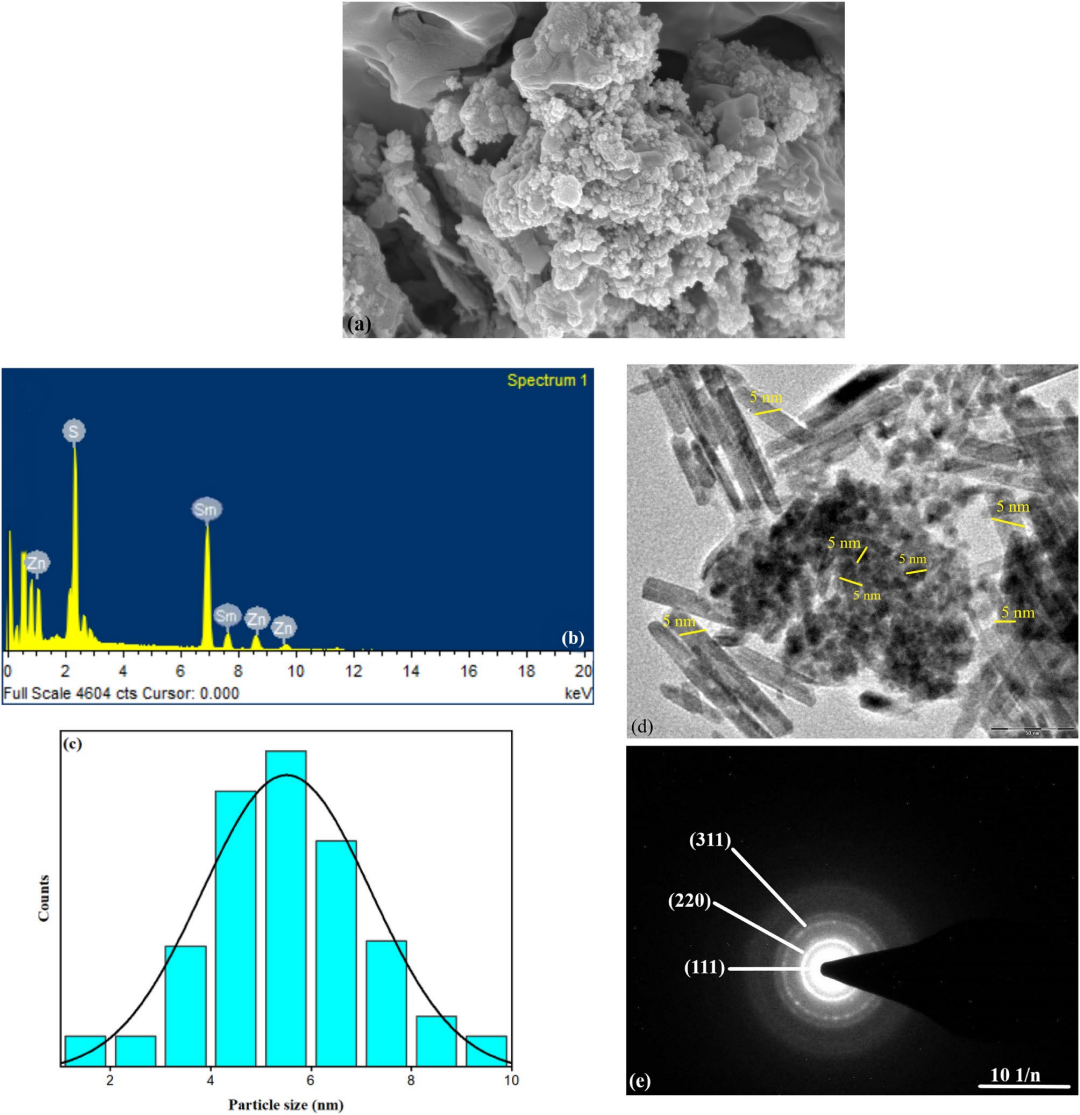
(**a**) SEM image of Sm^3+^-doped ZnS 9at. %. (**b**) Corresponding EDX spectra and (**c**) Average particle size of Sm^3+^-doped ZnS 9at. %. (**d**) TEM image of Sm^3+^-doped ZnS 9at. % and (**e**) SAED patterns of Sm^3+^-doped ZnS 9at. %.

SEM images and relative EDX spectra of 9at. % Sm^3+^-doped ZnS samples are shown in Fig. 2a,b. According to their morphology and microstructure, the samples are composed of various agglomerated particles that are largely irregular, non-uniform, and spherical. The term "agglomeration" describes the clustering or gathering of different particles. Since the samples are made up of various aggregated particles, it is clear that the zinc sulfide-doped samarium particles tend to collect rather than distribute evenly. The particles are described as having an erratic shape. This shows that the particles' shapes need to be more homogeneous and clearly defined. They might have uneven edges or rough surfaces as a result of many things, such as the synthesis process or the growth environment. The particles have a spherical form in addition to being classified as irregular. The synthesis technique, temperature, pressure, and other processing variables can all have an impact on the zinc sulfide doped with samarium's observable morphology and microstructure. The samples contain zinc, sulfide, and samarium elements, confirmed by the energy-dispersive X-ray examination. Atomic and weight percentages from the EDX are displayed in Table 2. The samples had an average size diameter of 5.15 nm, as shown in Fig. 2c. Figure 2d displays a TEM image of Sm^3+^-doped ZnS 9at. %. The estimated particle size from the TEM image was spherical as well as rod-shaped was found to be 5 nm. The nanometer (nm) range of this size measurement suggests that the sample's particles are quite small. The particles were both spherical and rod-shaped. Various synthesis processes or the presence of various crystallographic phases may have resulted in the coexistence of distinct particle morphologies. Especially in fields like catalysis, optics, and materials science, the size and form of nanoparticles can significantly affect both their characteristics and uses. Their size and form, as well as their crystalline structure and dopant dispersion, can all be affected by the precise synthesis process and conditions used to create these nanoparticles. Figure 2e displays the SAED patterns of Sm^3+^-doped ZnS nanoparticles as well as the locations of the three diffraction rings, which are the same as those seen in the (111), (220), and (311) diffraction of bulk ZnS^32^. From the selected area electron diffraction (SAED) pattern, the d-spacing is calculated. The first ring is 2.0 Å, the second is 1.6 Å, and the third is 1.2 Å.

**Table 2:** Atomic and weight percentages from the EDX.

Element	Weight	Atomic %
S	27.41	41.41
Sm	59.33	48.76
Zn	13.26	9.83
Total: 100.00		

**Figure 3:**
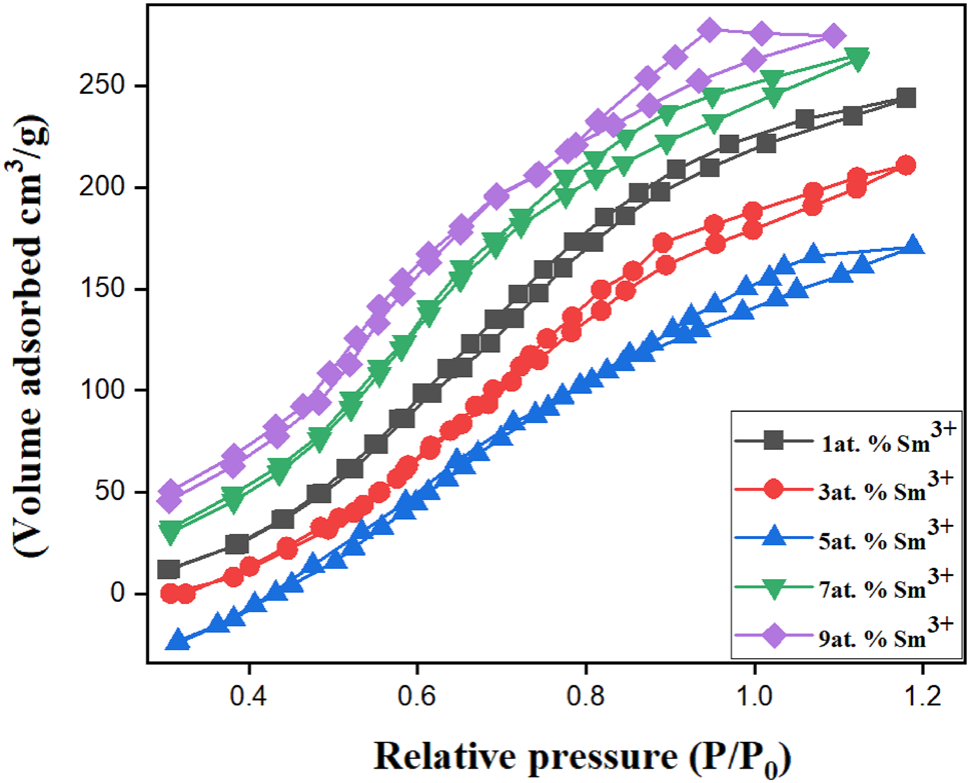
BET study of Sm^3+^ doped ZnS 1, 3, 5, 7, 9at. %.

The Brunauer Emmett Teller (BET) method is popular for calculating a particular surface area. The two primary factors in photodegradation are the vast surface area and the small particle size. Figure 3 shows the Sm^3+^-doped ZnS N2 absorption–desorption observations at 1, 3, 5, 7, and 9 at.%. The produced samples displayed type-IV H1 hysteresis loop isotherms, indicating mesoporous. Mesoporous (2 nm < pore size < 50 nm) materials produce the type IV isotherm^33^. The BET-specific surface areas were 54 m^2^/g, 72 m^2^/g, 88 m^2^/g, 113 m^2^/g, and 129 m^2^/g for 1, 3, 5, 7, and 9 at.% samples, respectively. The results of this BET analysis showed that Sm^3+^-doped ZnS 9at. % nanoparticles with large surface areas could efficiently promote electron and ion movement at the interface to improve dye degradation performance. This is a crucial observation because more active areas for chemical reactions exist when a surface area is more significant. The increased surface area of the Sm^3+^-doped ZnS nanoparticles promotes effective electron and ion transport at the interface between the nanoparticles and the dye molecules. The performance of dye degradation is improved due to the higher electron and ion movement^34^. The electrical structure and surface characteristics of the ZnS nanoparticles are probably affected by the Sm^3+^- dopant, which makes them better suited to enhanced electron transfer and facilitates the degrading process. Overall, the BET study shows that Sm^3+^-doped ZnS nanoparticles have properties that make them suitable catalysts for degrading dyes. Nanoparticles have proven to be highly effective in various applications, including dye degradation. This is mainly because nanoparticles possess a large surface area, enabling more efficient reactions. In addition, dopants like Sm^3+^ further enhance their performance, leading to superior results.

**Figure 4:**
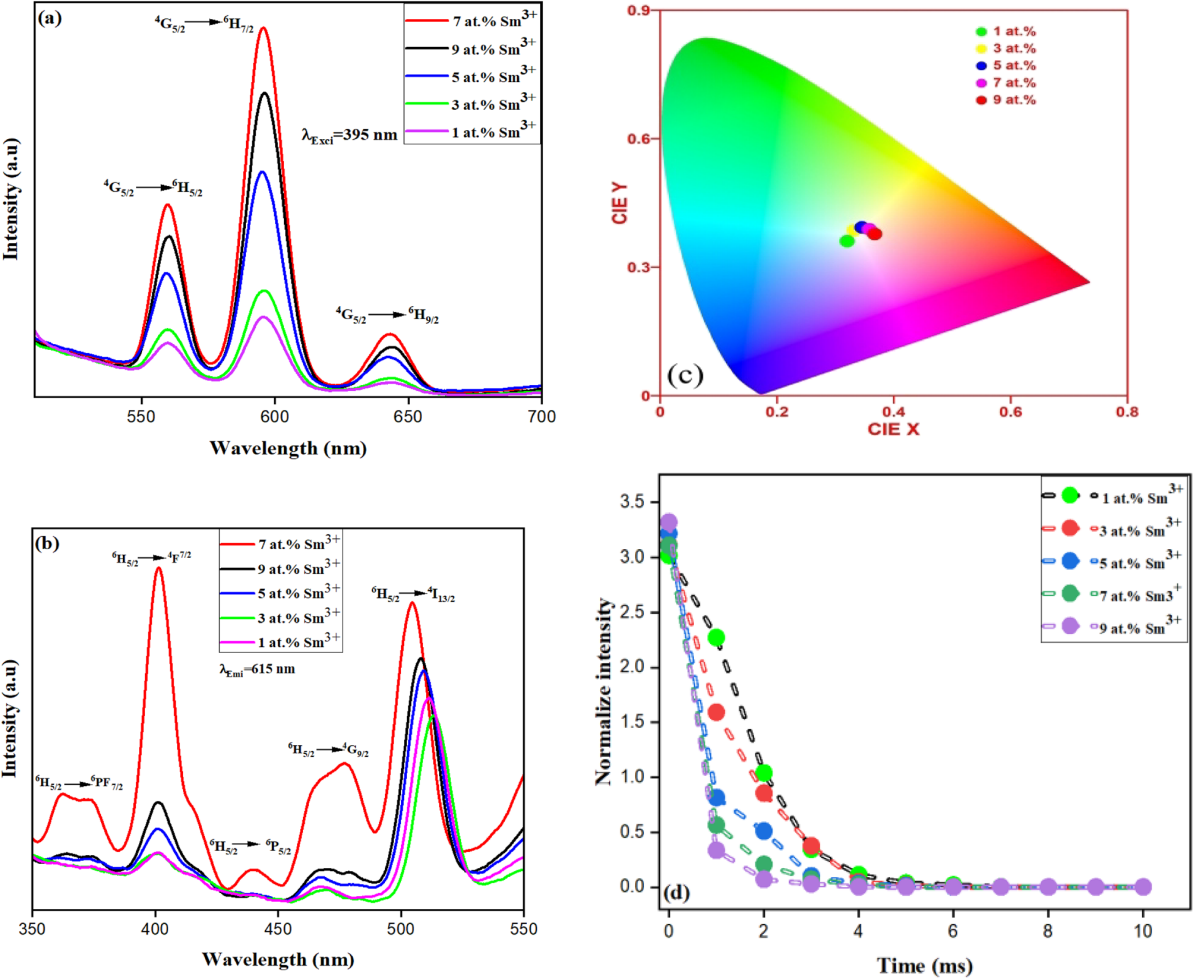
(**a**) Emission and (**b**) excitation spectra of Sm^3+^-doped ZnS 1, 3 5, 7, 9at. %. (**c**) CIE analysis, (**d**) Lifetime decay of Sm^3+^-doped ZnS 1, 3, 5, 7, 9at. %.

Figure 4a shows Sm^3+^-doped ZnS 1, 3, 5, 7, 9at. % emission peaks for photoluminescence spectra, which were measured between 500 and 700 nm. When excited at 395 nm, the peak positions are found to increase with an increase in Sm^3+^ concentrations up to 5 at. % and then decrease with a further increase in Sm^3+^ concentration, caused by the concentration quenching effect of Sm^3+^ induced by cross-relaxation. With increasing dopant concentration, there are more non-radiative transition events where energy is transferred from the initially absorbing impurity ions to comparable ions. A transfer to a quenching site (defect) may get implicated after many of these steps, further reducing the radiative process. Significant peaks in the emission spectra can be seen at wavelengths 559 nm (^4^G_5/2_ → ^6^H_5/2_), 595 nm (^4^G_5/2_ → ^6^H_7/2_), and 642 nm (^4^G_5/2 →_^6^H_9/2_), which correspond to the f-f transitions of Sm^3+^ in the 4f^5^ configuration, respectively. According to the selection rule, the peak at 559 nm (^4^G_5/2_ → ^6^H_5/2_) resulted from a pure dipole transition between an electric dipole (ΔJ =  ± 2) and a magnetic dipole (ΔJ = 0 and ± 1). While the transition (^4^G_5/2_ → ^6^H_7/2_) observed at 595 nm satisfies the selection rule for the magnetic dipole transition, it is attributed to the partially electric dipole and partially magnetic dipole transitions, whereas the electric dipole transition predominates. The peak (^4^G_5/2_ → ^6^H_9/2_) at 642 nm results from an electric dipole transition^35–37^. The impact of the crystal field on the transition between pure electric dipoles is significant indeed. It is also fascinating how it affects the transition between two pure electric dipoles. The Judd–Ofelt theory can be used to explain the local site symmetry around Ln^3+^ in a matrix^38^. Exception to their intensities, the peak profiles did not vary when Sm^3+^ concentrations changed, showing that Sm^3+^ had the exact site symmetry at all concentrations. Figure 4b displays the excitation spectra for the as-prepared Sm^3+^-doped ZnS 1, 3, 5, 7, and 9at. % obtained by monitoring when emitted at 615 nm. The spectra comprise a broad band between 350 and 550 nm. The electronic transition from Sm^3+^ ground state (^6^H_5/2_) to various excited energy levels produces spectra with sharp, narrow peaks. They can be positioned at 367 nm (^6^H_5/2_ → ^6^P_7/2_), 401 nm (^6^H_5/2_ → ^4^F_7/2_), 439 nm (^6^H_5/2_ → ^6^P_5/2_), 476 nm (^6^H_5/2_ → ^4^G_9/2_), and 501 nm (^6^H_5/2_ → ^4^I_13/2_). Due to Sm^3+^ f-f transitions, it can be seen that the broad absorption band's intensity is more significant than those narrow peaks, indicating that energy has been transferred from the host lattice to Sm^3+^^39^. These results agree with the earlier publications on the photoluminescence features of Sm^3+^^40,41^. The luminescence properties of ZnS are changed by doping with Sm^3+^ ions, which introduce luminous centers into the crystal structure. The characteristic energy level transition in Sm^3+^ ions results in luminescence at particular wavelengths. The excitation wavelength, Sm^3+^ doping concentrations, and luminescence intensity of Sm^3+^ doped ZnS will all affect the emission spectra and luminescence intensity. Applications for the luminescence produced by Sm^3+^ doped ZnS include optoelectronics, display technology, luminescence devices, etc^16^.

For applications involving photoluminescence, CIE colour coordinates are important. Figure 4c Displays the CIE chromaticity curve for synthesized Sm^3+^-doped ZnS at 1, 3, 5, 7, and 9at. %. This diagram showed that when triggered at 395 nm, the generated samples emit a hue that is almost entirely white hue. Figure 4d shows the emission decay curves for Sm^3+^-doped ZnS samples at 1, 3, 5, 7, and 9at. %. The bi-exponential function can be used for precisely fitting the entire decay curve as given in Eq. (6)^42^:

6 $${\text{I}} = {\text{I}}_{1} \exp \left( { - {\text{t}}/\uptau _{1} } \right) + {\text{I}}_{2} \exp \left( { -\uptau /\uptau _{2} } \right).$$

I_1_ and I_2_ represent the equivalent decays of intensities at two different points in time ‘t’. It displays the values of two lifetimes: slow time τ_2_ and quick time τ_1_. The key variables influencing the behaviour of bi-exponential decay are the number of luminescence centers, energy transfer from the donor, the unequal distribution of dopant ions on the host lattice, and impurities.

I_1_ and I_2_ represent equivalent decays of intensities at different points in time 't'. The values of slow time τ_2_ and quick time τ_1_ play a crucial role in determining the behavior of bi-exponential decay. Factors such as the number of luminescence centers, energy transfer from the donor, unequal distribution of dopant ions on the host lattice, and impurities can also impact the behavior of bi-exponential decay. The typical luminescence lifespan can be calculated using the following equation^43^.

7 $$*\uptau = I_{1}\uptau _{1}^{2} + I_{2}\uptau _{2}^{2} /I_{1}\uptau _{1}^{2} + I_{2}\uptau _{2}$$

The calculated average life times of Sm^3+^-doped ZnS at concentrations 1, 3, 5, 7, 9at. % were found to be 1.88, 1.71, 1.89, 1.52, 1.49 ms respectively. According to speculation, the concentration quenching effect is what causes the life time value to decrease with increasing Sm^3+^ concentration.

**Figure 5:**
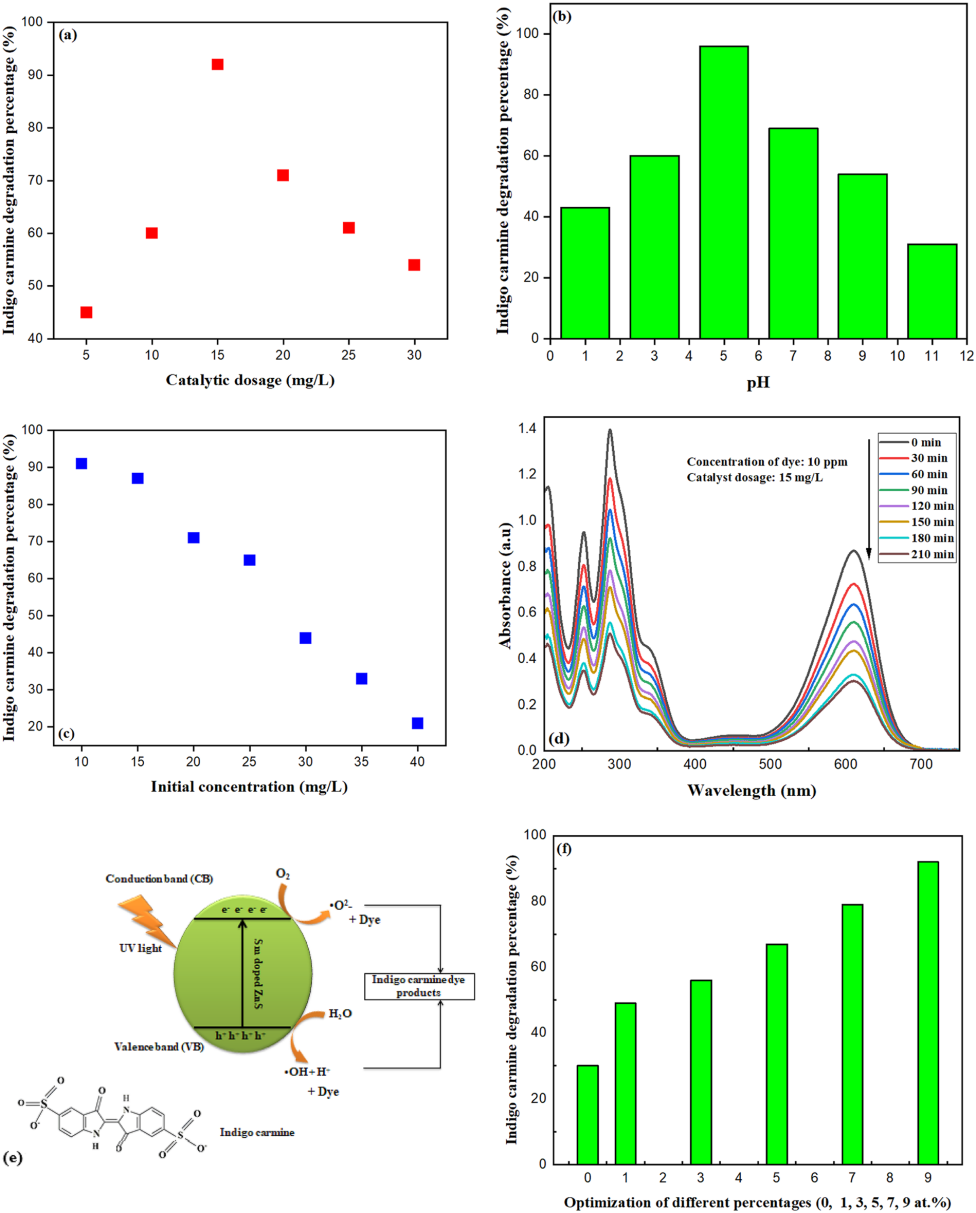

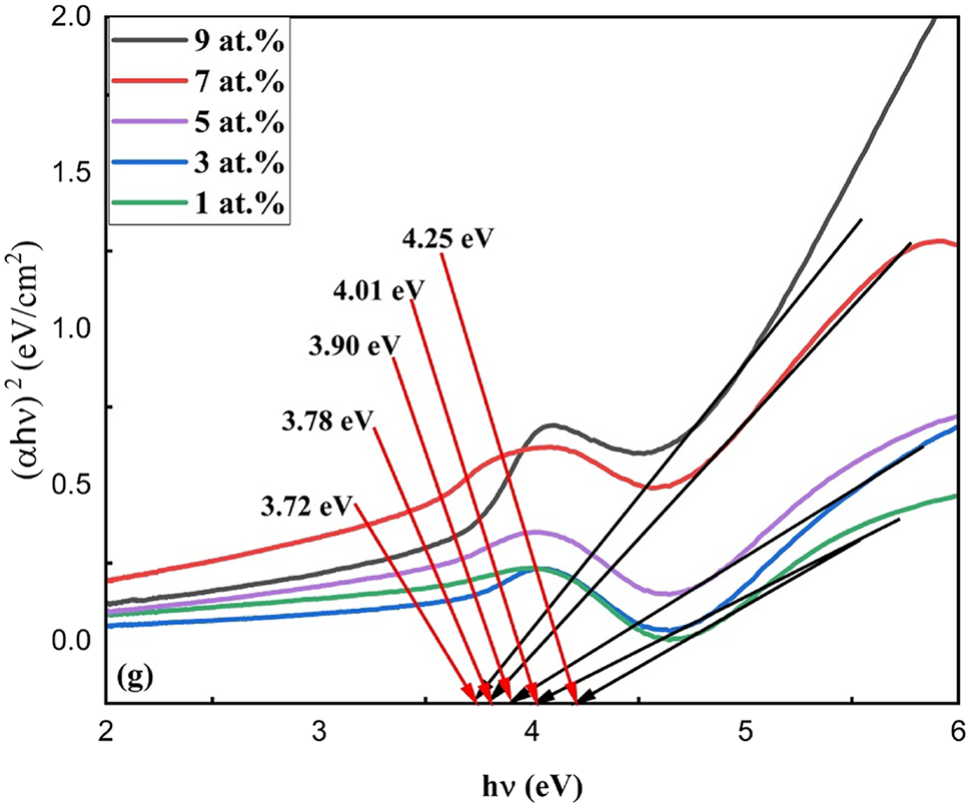
(**a**) Amount of catalytic dosage on the degradation of indigo carmine. (**b**) Effect of different pH concentration on the degradation of indigo carmine. (**c**) Initial concentration study on the degradation of indigo carmine. (**d**) Degradation of Indigo Carmine within 210 min. (**e**) Structure of indigo carmine and photocatalytic degradation. (**f**) Optimization of different atomic percentage concentration on the degradation of indigo carmine. (**g**) Tauc plot of Sm^3+^-doped ZnS different percentage.

Nanocatalyst doses ranging from 5 to 30 mg/L are exposed to light for 210 min. Due to an increase in active sites, the rate of decolourisation increases to its maximum degrading efficiency in the presence of 15 mg/L of nano-photocatalyst. However, as the loading was increased above the optimal level, the decolourisation rate was reduced due to the increased opacity of the suspension samples and, as a result, increased light scattering, as seen in Fig. 5a. The pH range where the solution can degrade indigo carmine was found to be 5.3. A diluted hydrochloric acid or potassium hydroxide solution was used to modify the pH value when necessary. Different solutions were created with pH values ranging from 1, 3, 5, 7, 9, and 11. Following 210 min of photocatalytic irradiation, the degradation efficiencies were examined. The effectiveness of the indigo carmine solution's degradation decreased as the pH level of the reaction solution increased, as shown in Fig. 5b. When the pH was 5, degradation efficiency was higher, but when it was 11, it reduced to 30.45%, indicating that the optimal pH for degradation was found to be pH 5. Since indigo carmine is a synthetic dye, its stability and chemical properties, like those of many other dyes, may be pH-dependent. The dye molecule may be more vulnerable to breakdown at pH 5 due to several mechanisms, including hydrolysis, oxidation, or reduction. The dye molecule may be more likely to degrade at this ideal pH. Indigo carmine can be broken down more quickly at pH 5 because some enzymes or catalysts may be more active at that level. The dye may be more soluble or more likely to form aggregates more vulnerable to deterioration processes. The molecule may be more prone to photodegradation, where light energy triggers chemical processes that result in dye deterioration.

Dye solutions have been produced in concentrations ranging from 10 to 40 (ppm). 10 ppm shows the best degradation percentage. The decreasing efficiency decreased as the starting concentration rose, as indicated in Fig. 5c, and the tendency accelerated as the concentration increased. As the concentration increased, more photons would be absorbed, limiting light transmission. As a result, less light was able to penetrate deeply, limiting the nanoparticle's potential to catalyze the processes. The ability of a chemical to deteriorate at varied doses over time was investigated. Figure 5d shows the indigo carmine degradation in 210 min at pH 5 with concentrations of 10 ppm and 15 mg/L of catalytic dose. Indigo carmine absorbs the most light in the visible spectrum at a wavelength peak of 615 nm. Indigo carmine absorbs some wavelengths of visible light and reflects or transmits others when it is in contact with the light. In this instance, it primarily absorbs light in the blue and green portions of the spectrum, giving it a blue hue. The absorption at 615 nm is defined by the conjugated system of aromatic rings' molecular structure and electronic transitions. The energy needed to move molecules electrons from their lower-energy (ground) states to their higher-energy (excited) states is represented by this absorption peak. Figure 5e provides information on the structure of indigo carmine and the potential degradation mechanism. The photocatalytic performance of ZnS for the degradation of dyes concerning this work is given in Table 3. Dye degradation is a systematic process with a well-defined course and intuitive mechanism. ZnS has an energy band structure with a low valence band (VB) filled with electrons and a high empty energy conduction band (CB). It has been demonstrated that when the energy of the radiation exceeds the band gap width of ZnS, matched holes (h^+^) grow in the valence band (VB), creating a “electron hole” pair. In contrast, excited electrons in the valence band (VB) can jump into the conduction band (CB)^44^. According to Eqs. (8–14), superoxide ions (O_2_^⋅^) are created when absorbed oxygen (O_2_) and conduction band electrons (CB) interact under the conditions of irradiation. These reactive oxygen species (O_2_^⋅^, OH⋅, and H_2_O_2_) have an exceptionally high redox capacity along the whole photocatalytic reaction pathway. They are capable of breaking down large organic molecules into smaller molecules, more environmentally friendly molecules like CO_2_ and H_2_O. In order to speed up chemical reactions that break down dye molecules, photocatalytic degradation of dyes uses light to activate catalyst materials. The bandgap energy of the catalyst material may change due to doping. Doping enables the material to absorb a wider range of light, including visible light, by reducing the bandgap. The photocatalytic process depends on the photogenerated electron–hole pairs' recombination rate, which doping can lower. Reduced recombination means that more of these charge carriers can interact with dye molecules in redox processes, which increases the effectiveness of dye degradation. Dopant atoms can increase the number of active sites and change the catalyst's surface characteristics, facilitating dye molecule adsorption and subsequent reactions. More dye molecules may interact with the catalyst's active sites as a result, increasing catalytic activity. The degradation of indigo carmine using pure, 1, 3, 5, 7 and 9 at.% is shown in Fig. 5f, and it demonstrates that 9at.% was the best for dye degradation, demonstrating the significant influence that doping has on photocatalytic activity. Doping a catalyst can considerably boost its performance in dye degradation, particularly in photocatalysis, by extending its light absorption range, boosting charge separation and transport, increasing active sites for adsorption, and fine-tuning. These advancements are essential for wastewater treatment and environmental remediation procedures that are more effective and sustainable. The nanoparticles' yield as a percentage was above 88%. The highest yield is achieved at 9 at. %; it also demonstrates a greater advantage in the degradation of nanoparticles and is most effective in the degradation of indigo carmine. The purity of the starting ingredients, the effectiveness of the synthesis process, and the methods used for purification can all impact on the yield of nanoparticles. Due to the difficulties in regulating and isolating nanoparticles in a highly pure state, nanoparticles frequently have lower yields than bulk materials. The abovementioned processes demonstrate that photocatalytic degradation is possible when soluble oxygen and H_2_O are present. There have been other reports of similar methods for the photocatalytic destruction of dyes^44^. As a result, ZnS nanoparticles have the potential to purify them, which causes photocatalysis to destroy indigo carmine dye. The photodegradation mechanism is represented by the following steps:

8 $${\text{ZnS}} + {\text{h}}\upnu \to {\text{e}}^{ - } + {\text{h}}^{ + }$$

**Table 3:** Comparision of the photocatalytic performance of ZnS for the degradation of dyes with reference to this work.

Sample	Light	Catalyst (mg)	Concentration (ppm)	Time (min)	Dye	Degradation %	Rate constant (min^−1^)	References
ZnS 1 ZnS 2	Mercury lamp (70W)	5	10	180	Methylene Blue	55.97 65.92	0.00594	^45^
Chitosan-ZnS	UV lamp	30	30	100	Acid black-234	96	0.01464	^46^
Chitosan-ZnS	UV lamp	30	30	165	Acid brown-98	92	0.04096	^46^
ZnS 1	Sodium lamp OSRAM VIALOX (80W)	5	5	180	Green rhodamine B	70.60	0.0061	^47^
ZnS 2	Sodium lamp OSRAM VIALOX (80W)	5	5	180	Green rhodamine B	88.80	0.0116	^47^
ZnS 3	Sodium lamp OSRAM VIALOX (80W)	5	5	180	Green rhodamine B	98.51	0.0208	^47^
ZnS:Sm^3+^	UV light	15	10	210	Indigo carmine	93	0.0261	This work

Zinc sulfide (ZnS) absorbs light energy (hѵ) and undergoes photoexcitation. This process generates an electron (e^−^) and a positive hole (h^+^).

9 $${\text{e}}^{ - } + {\text{O}}_{2} \to {\text{O}} \cdot _{2}^{ - }$$

The generated electron (e^−^) reacts with oxygen (O_2_) to form superoxide anion (O·_2_^−^).

10 $${\text{O}}_{2}^{{ \cdot - }} + {\text{h}}^{ + } \to {\text{HO}} \cdot _{2}$$

The positive hole (h^+^) reacts with superoxide anion (O·_2_^−^) to form hydroperoxyl radical (HO⋅_2_).

11 $${\text{HO}}_{{ \dot 2}} + {\text{e}}^{ - } \to {\text{h}}^{ + } \to {\text{H}}_{2} {\text{O}}_{2}$$

The hydroperoxyl radical (HO⋅_2_) reacts with the generated electron (e^−^) to form a positive hole (h^+^), leading to the production of hydrogen peroxide (H_2_O_2_).

12 $${\text{H}}_{2} {\text{O}} + {\text{e}}^{ - } \to {\text{OH}}^{ \cdot } + {\text{OH}}^{ - }$$

Water (H_2_O) reacts with the generated electron (e^−^) to produce hydroxyl radical (OH^⋅^) and hydroxide ion (OH^−^).

13 $${\text{h}}^{ + } + {\text{OH}}^{ - } \to {\text{OH}}^{ \cdot }$$

The positive hole (h^+^) reacts with hydroxide ion (OH^−^) to form hydroxyl radical (OH^⋅^).

14 $${\text{OH}}^{ \cdot } /{\text{O}}_{2}^{ - } + {\text{Indigo}}\;{\text{carmine}}\;{\text{dye}} \to {\text{degradation}}\;{\text{products}}$$

Hydroxyl radicals (OH^⋅^) or superoxide anions (O·_2_^−^) react with Indigo carmine dye, leading to the degradation of the dye and the formation of various degradation products.

Figure 5g shows the UV–Vis absorption spectra of different atomic percentages. The energy of the band gap (Eg) of ZnS nanoparticles can be calculated from the UV–Vis spectra by creating a Tauc plot of (hѵα)^2^ versus (hѵ) and extrapolating the linear portions of the curves to the energy axis:

$$\alpha h\nu = B\left( {h\nu {-}Eg} \right)^{1/2}$$

where *Eg* is the direct band gap energy, *hѵ* is the photon energy,* α* is the absorption coefficient, and *B* is a constant. The calculated bandgap was found to be 4.25, 4.01, 3.90, 3.78 and 3.72 eV for 1, 3, 5, 7, and 9 at.% respectively. Depending on the concentration and type of doping, adding samarium (Sm^3+^) to zinc sulfide (ZnS) might change its bandgap. Samarium can introduce energy levels within the bandgap by increasing doping concentrations, effectively reducing the bandgap. This process is referred to as “bandgap narrowing”. Samarium introduces impurity states in these conditions, decreasing the energy required for electron excitation and facilitating electron migration from the valence band to the conduction band. Samarium concentration and material crystal structure will determine the exact impact on the bandgap.

An adsorption isotherm describes the relationship between the amount of dye adsorbed by the adsorbent at equilibrium and the equilibrium dye concentration in the solution. For assessing the viability of the adsorption process as a single operation, adsorption equilibrium provides essential physiochemical information. This isotherm accounts for the effects of numerous dye/adsorbent interactions that are indirect, and it predicts that these interactions will cause a linear reduction in the heat of adsorption with increasing molecule-by-molecule coverage of the layer. Adsorption equilibrium gives critical physiochemical data for assessing the adsorption process's applicability as a single operation. The Freundlich and Langmuir isotherm models examined the relationship between solution concentration and dye adsorption. The isotherm's best fit was determined using a linear regression value^48^.

Freundlich Isotherm:

The adsorption on heterogeneous surfaces is explained by this isotherm, and the data frequently suit the provided equation^49,50^.

15 $$q_{e } = K_{f} C_{e} \frac{1}{n}$$

The Freundlich isotherm has the following logarithmic form:

16 $$\log q_{e} = \log K_{f} + \frac{1}{n}\log C_{e}$$

**Figure 6:**
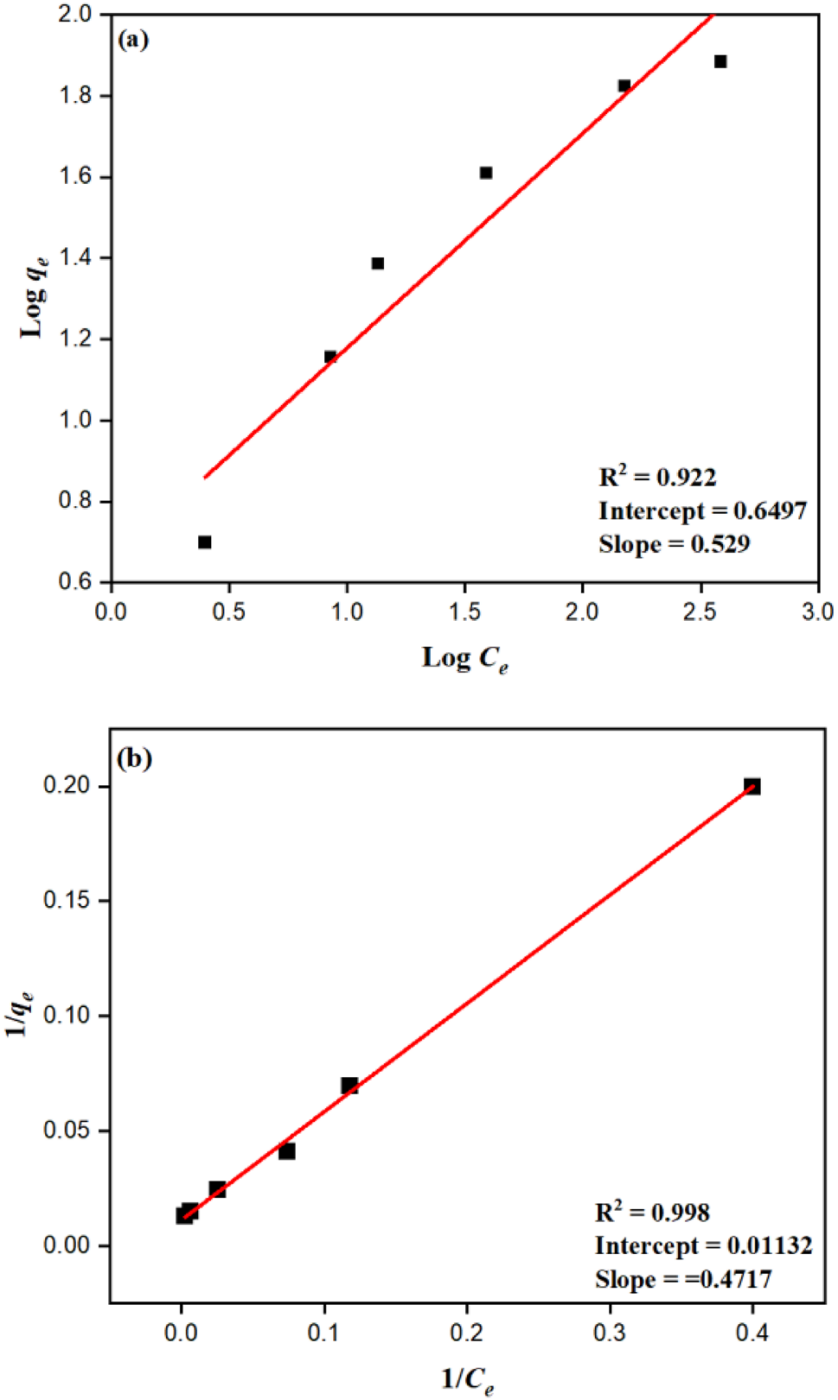
(**a**) Freundlich isotherm model and (**b**) Langmuir isotherm model of degradation of Indigo carmine.

The equilibrium amount of dye adsorbed per gram of adsorbent is indicated by *q*_*e*_ (mg/g), and the equilibrium adsorbate concentration is denoted by the symbol *C*_*e*_ (mg/L). The slope and intercept of the line shown in Fig. 6a can be used to determine the sorption intensity (g/L), or 1/n and $K_{f}$ (Freundlich constant) where R^2^ was found to be 0.922.

Langmuir Isotherm:

The equation can be used to calculate this isotherm, which provides data on the production of monolayers on homogeneous surfaces:

17 $$q_{e} = \frac{{K_{L} C_{e} }}{{1 + aLC_{e} }}.$$

The equation can be written as follows in linearized form:

18 $$\frac{{ C_{e} }}{{ q_{e} }} = \frac{1}{{K_{L} }} + \frac{{aLC_{e} }}{{K_{L} }}$$

Langmuir isotherm constants are K_*L*_ and a_*L*_; the concentration at equilibrium (mg/L) is represented by $C_{e}$; *q*_*e*_, which is the adsorbed amount (mg/g), K_*L*_/a_*L*_ is the numerical equivalent of the theoretical monolayer capacity (Q_o_). C*e*/*qe* versus C*e* was used to depict the Langmuir adsorption isotherm and a line with a 0.998 R^2^ correlation coefficient was found given in Fig. 6b. Table 4 shows the calculated parameters for the Langmuir and Freundlich isotherm models. The Langmuir isotherm model may be the best-fitting model for the sorption of indigo carmine dye, according to the estimated correlation value R^2^ shown in Table 4.

**Table 4:** Calculated parameters for Langmuir and Freundlich isotherm models for degradation of indigo carmine dye at 10ppm concentration.

Isotherm models	Parameters	Values
Langmuir isotherm	q_max_ (mg/g)	88.339
	K_*L*_ (l/g)	2.119
	R	106.99
	R^2^	0.998
Fruendlich isotherm	1/n	0.529
	K_f_ (mg/g)^1/n^	4.463
	R^2^	0.922

The primary step in many separation and purification procedures is adsorption. The molecules or ions from a solution adhere to the surface of a solid or liquid adsorbent. Mathematical representations known as kinetic models are used to explain the adsorption rate. It is predicated on the idea that the rate of adsorption is inversely proportional to the solute concentration. In other words, the rate of adsorption increases with increasing solute concentration. The pseudo-first-order model may not be applicable in all situations but is appropriate for a system where adsorption is largely a physical process. Another kinetic model used to describe adsorption is the pseudo-second-order model. It is predicated on the idea that the adsorption rate is inversely proportional to solute concentration squared. This model can better fit systems where adsorption involves chemical interactions between the solute and the adsorbent when dealing with heterogeneous sorbents and chemical sorption. Experimental data on the degradation of indigo carmine is gathered and analyzed using these models to shed light on the adsorption mechanism and validate the kinetic models. The system will influence model selection under study, the type of adsorption, and the adsorbent's characteristics. The pseudo-first-order equation is expressed as^51^:

19 $$\log \left( {qi - qt} \right) = log_{qe} - \frac{{k_{1} }}{2.303}t$$

And *q*_*e*_ is the catalyst amount (mg/g) at equilibrium. *q*_*t*_ is the catalyst quantity (mg/g) at any given time (min^−1^), *q*_*e*_ is the catalyst quantity (mg/g) at equilibrium, and *k*_1_ is the first-order reaction rate constant.

The pseudo-second-order model is based on the hypothesis that chemisorption, which involves valence forces through the sharing or exchange of electrons between adsorbent and adsorbate, may be the rate-limiting phase. The pseudo-second-order equation is represented as follows^52–54^:

20 $$\frac{t}{{q_{t} }} = \frac{t}{{q_{e} }} + \frac{1}{{kq_{e}^{2} }}$$

**Figure 7:**
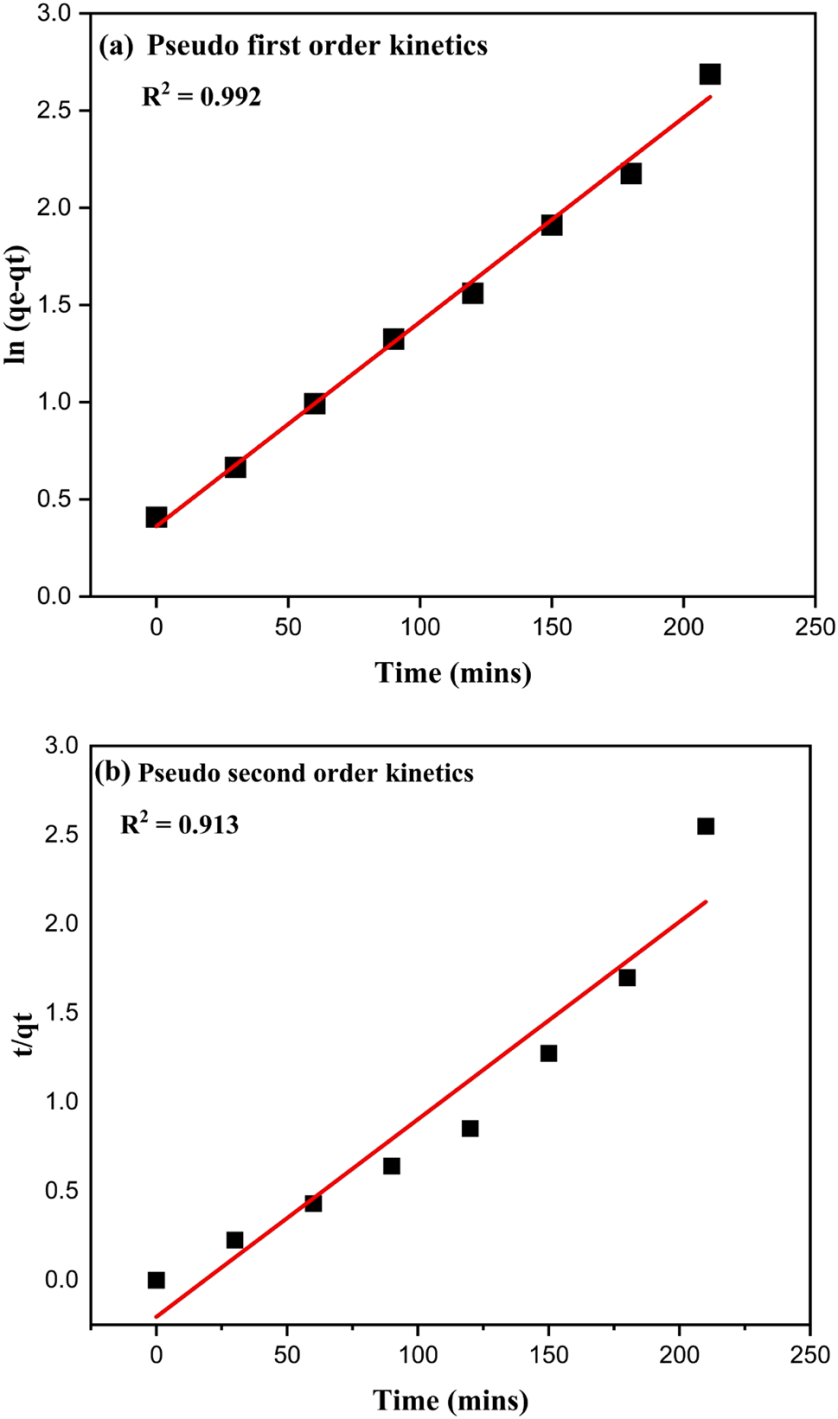
(**a**) Pseudo first kinetic order model, (**b**) Pseudo second order kinetic model.

The catalyst amount (mg/g) at a specific time (min^−1^) is given by *q*_*t*_, *q*_*e*_ is the catalyst amount (mg/g) at equilibrium, and *k*_2_ is the pseudo-second-order reaction rate constant. The rate constant for the first order kinetic (*k*_1_) was calculated using a linear plot of time (*t*) vs. log (*q*_*e*_–*q*_*t*_), where *q*_*e*_ and *q*_*t*_ represent the quantity of dye adsorbed (mg/g) at equilibrium and time (t), respectively. The second-order kinetic (k_2_) rate constant was calculated using the linear time vs. *t/qt* plot shown in Fig. 7a,b. Table 5 shows the rate constant and R^2^ values, which follow pseudo-first-order kinetics. The pseudo-first-order kinetic model's R^2^ value is considerably more significant than the second-order kinetic model's. As a result, the adsorption of indigo carmine dye using Sm^3+^-doped ZnS nanoparticles is well correlated using the pseudo-first-order kinetic model.

**Table 5:** Calculated values of R^2^ and rate constant for pseudo first and second kinetics models for the degradation of indigo carmine dye at a concentration of 10 ppm.

Models	R^2^ value	Rate constant
Pseudo first order	0.992	k_1_ = 0.0261 min^−1^
Pseudo second order	0.913	k_2_ = 0.0122 g/mg × min

**Figure 8:**
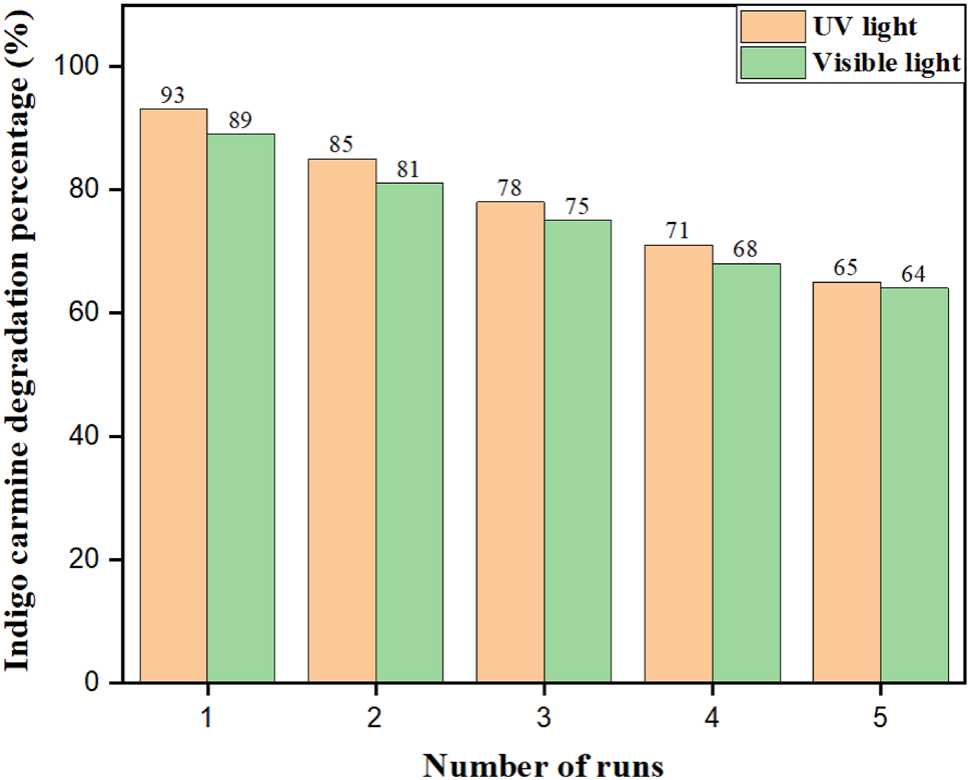
Reusability of a catalyst.

As a result of the repeated usage of nanophotocatalysts for the degradation of indigo carmine dye, the reusability of the catalysts has been studied, as shown in Fig. 8. The results show that reusability is good for up to five consecutive cycles. The nanophotocatalyst was collected by filtration after the first photodegradation cycle. After being thoroughly cleaned with deionized water, it was dried in an oven for an entire night at room temperature. The experiment was conducted both in the presence of UV and visible light. It clearly shows that in UV light, photocatalytic degradation decreases from 93 to 65%, and in visible light, it decreases from 89 to 64%. The photocorrosion effect is the cause of this decline in photocatalytic effectiveness. A material oxidatively degrades in the presence of light, which is known as the photocorrosion effect. Under UV light and sunlight, ZnS undergoes photocorrosion.

## Conclusion

In this study, the focus was on the synthesis and characterization of Sm^3+^ doped ZnS nanoparticles using the reflux method. The research team investigated several properties of the nanoparticles, including kinetics, structural, chemical, optical, isotherms, luminescence, and photocatalytic properties. The results indicated that Sm^3+^ doping had a positive impact on the luminescence and photocatalytic properties of the ZnS nanoparticles. The maximum photocatalytic activity has been observed in 9 at% Sm^3+^-doped ZnS nanoparticles, which were used for further evaluation. The best optimum parameters for removing dye were found to be 15 mg/L of catalyst, 210 min of reaction time, pH 5, and an initial concentration of 10 ppm. All the synthesized samples exhibited a zinc cubic phase, as observed in the XRD pattern. The average particle size calculated from the SEM image was found to be 5.15 nm. According to photoluminescence studies, when excited at 395 nm, the emission spectra can be seen at wavelengths 559 nm (^4^G_5/2_ → ^6^H_5/2_), 595 nm (^4^G_5/2_ → ^6^H_7/2_), and 642 nm (^4^G_5/2 →_^6^H_9/2_), which correspond to the f-f transitions of the Sm^3+^ in the 4f^5^ configuration, respectively. The results of CIE diagram showed that the nanoparticles can be highly useful for photonic applications. Five catalyst runs could be successfully retrieved without the catalyst experiencing a significant loss of activity. The reaction had pseudo-first kinetics, and the Langmuir model best matched the isotherm data. As a result of improved charge separation and decreased recombination, Sm^3+^-doped ZnS can increase the photocatalytic degradation efficiency of indigo carmine. Overall, the study suggests that Sm^3+^-doped ZnS nanoparticles can be an effective and promising method for removing coloured effluents, with significant potential for reusability and luminescent applications.

## Methods

Sodium sulfide (98.0% purity) Na_2_S·9H_2_O Alfa-Aesar, zinc acetate dihydrate (98.0–101.0% purity) Alfa Aesar (CH_3_COO)_2_ Zn·2H_2_O, and Samarium(III) nitrate hexahydrate (99.9% purity) Sm(NO_3_)_3_.6H_2_O Alfa-Aesar were used in the reflux method to synthesize the nanoparticles of Sm^3+^ doped ZnS.

### Procedure

Sm^3+^-doped zinc sulfide nanoparticles with varying concentrations (1, 3, 5, 7, and 9at. %) were synthesized using the reflux process. Samarium and ZnS were mixed and stirred at room temperature for 45 min. Drop by drop, sodium sulfide was added to the aforementioned solution. The entire solution was then refluxed at 160 °C for three hours. The resultant residue was washed with acetone, centrifuged at 7000 rpm and dried at 100 °C for 5 h. Various doping concentrations of Sm^3+^ doped zinc sulfide nanoparticles were synthesized using the same process.

### Formation of nanoparticles

Samarium is incorporated as a dopant into the ZnS lattice by carefully adding samarium nitrate to the zinc precursor solution. The doping concentration is determined by the amount of samarium introduced, and it can be adjusted to modify the characteristics of the nanoparticles. The reaction is usually conducted at elevated temperatures under controlled conditions using a mixture of zinc and sulfur precursor solutions. As a result, ZnS nanoparticles start to form. During the nucleation process, the doped samarium ions are integrated into the ZnS lattice, creating Sm^3+^-doped ZnS nanoparticles. The nanoparticles keep expanding as the reaction goes on. Stabilizing agents or surfactants may be applied to manage the nanoparticles' size, shape, and aggregation. The reaction mixture is removed from the Sm^3+^-doped ZnS nanoparticles, frequently by centrifugation or filtration, and the nanoparticles are cleaned and purified to get rid of any unreacted precursors or byproducts.

### Characterization

Different characterization techniques were used to characterize the prepared samples. Using Rigaku SmartLab, the crystal structure and phase composition were discovered using XRD. The SEM image was obtained using a Carl Zeiss Supra 55 FESEM. Using the FEI TECNAI G2 F20 S-TWIN, TEM images were captured. Shimadzu's IRAffinity-1S was used to collect FTIR data. A fluorescence spectrometer F-7000 with a (150 W) Xenon lamp was used to evaluate the photoluminescence characteristics. Novatouch LX Quantachrome was used to collect BET data. A wavelength range of 190 to 1100 nm evolution 220 UV visible spectrophotometer was used to evaluate the photocatalytic properties.

### Photocatalytic degradation procedure and analysis

The photocatalytic activity was investigated by observing the photodegradation of indigo carmine. Different dye concentrations (between 10 and 40 ppm) with catalytic doses (5–30 mg/L) and pH ranges (1, 3, 5, 7, and 11) were studied. Each reaction was stirred with a magnetic stirrer for 60 min to ensure that the catalyst suspension was uniform throughout the reaction for the degradation tests and exposed to UV light Philips PL-S 92/WP BLB. Samples were taken regularly, filtered using Millipore membrane filters, and centrifuged to get rid of any undissolved materials to calculate the percentage of dye degradation. A small amount was extracted with a syringe and filtered through a 0.45 mm hole millipore syringe filter. The composite nanoparticles were repeatedly cleaned with distilled water after each experiment in order to allow for reuse. Using a UV–visible spectrophotometer, the UV absorbance in the 200–700 nm region was recorded at certain intervals (every 30 min), and the dye concentration was then calculated. Accordingly, the photodegradation efficiency was determined using the formula ^51^:

The photodegradation efficiency was calculated using the formula provided in the text, which considers the starting concentration of the dye and the concentration at a given time. UV–visible spectrophotometer was used to measure the UV absorbance in the 200–700 nm region at specific intervals of 30 min, and the dye concentration was calculated accordingly. This allowed for the determination of the photodegradation efficiency of the composite nanoparticles. The formula used for this calculation was efficiency (%) of photodegradation = (C_i_ − C_t_)/C_i_ × 100, where C_i_ is the starting concentration of the dye and C_t_ is the concentration at time ‘t’. By evaluating the photodegradation efficiency, the durability and usability of the photocatalytic catalysts were determined.

$${\text{Efficiency}}\;\left( \% \right)\;{\text{of}}\;{\text{photodegradation:}}\;\frac{{C_{{i - }} C_{t} }}{{C_{i} }} \times 100$$

where $C_{i}$ denotes the starting concentration and $C_{t}$ denotes the concentration at time ‘t’. After each cycle of degradation, the photocatalytic catalyst's durability and usability were evaluated. The catalyst was filtered out and repeatedly rinsed with distilled water before the dyes were photodegraded under the same circumstances.

## Data Availability

Data are available from the authors upon reasonable request and with the permission of the corresponding author.

## Abbreviations

XRD: X-ray diffraction
FTIR: Fourier Transform Infrared Spectroscopy
SEM: Scanning Electron Microscopy
SAED: Selected area electron diffraction
EDX: Energy dispersive X-ray analysis
TEM: Transmission electron microscopy
BET: Brunauer–Emmett–Teller
PL: Photoluminescence
UV: Ultraviolet spectroscopy
AOP: Advanced oxidation process
FWHM: Full width half maximum
ROS: Reactive oxygen species
VB: Valence band
CB: Conduction band
